# Evaluation of the Diagnostic Value of Contrast-Enhanced Voiding Urosonography with Regard to the Further Therapy Regime and Patient Outcome—A Single-Center Experience in an Interdisciplinary Uroradiological Setting

**DOI:** 10.3390/medicina57010056

**Published:** 2021-01-09

**Authors:** Constantin A. Marschner, Vincent Schwarze, Regina Stredele, Matthias F. Froelich, Johannes Rübenthaler, Thomas Geyer, Dirk-André Clevert

**Affiliations:** 1 Department of Radiology, University Hospital, LMU Munich, 81377 Munich, Germany; vincent.schwarze@med.uni-muenchen.de (V.S.); johannes.ruebenthaler@med.uni-muenchen.de (J.R.); thomas.geyer@med.uni-muenchen.de (T.G.); dirk.clevert@med.uni-muenchen.de (D.-A.C.); 2 Department of Urology, Ludwig-Maximilians-University Munich, 81377 Munich, Germany; regina.stredele@med.uni-muenchen.de; 3 Department of Clinical Radiology and Nuclear Medicine, University Medical Centre Mannheim, 68167 Mannheim, Germany; matthias.froelich@umm.de

**Keywords:** vesicoureteral reflux, contrast-enhanced urosonography, voiding cystourethrography, endoscopic treatment, laparoscopic treatment

## Abstract

*Background and Objectives*: Vesicoureteral reflux (VUR) describes a common pediatric anomaly in pediatric urology with a prevalence of 1–2%. In diagnostics, in addition to the gold standard of voiding cystourethrography (VCUG), contrast-enhanced urosonography (ceVUS) offers a radiation-free procedure, which, despite its advantages, is not yet widely used. In the present single-center study, subsequent therapeutic procedures and outcomes after ceVUS of 49 patients were investigated. The aim of the study is to investigate the efficacy of ceVUS with the intention of broader clinical implementation. *Materials and Methods*: Between 2016 and 2020, 49 patients were retrospectively included and received a ceVUS to evaluate VUR. With a distribution of 47:2 (95.9%), a clear female predominance was present. The age of the patients varied between 5 months and 60 years at the time of ceVUS. All examinations were all performed and subsequently interpreted by a single experienced radiologist (EFSUMB level 3). *Results*: Compared to intraoperative findings, ceVUS shows a sensitivity of 95.7% with a specificity of 100%. Allergic reactions to the contrast medium could not be observed. *Conclusion*: With its high sensitivity and intraoperative validation, ceVUS offers an excellent alternative to VCUG, the gold standard in the diagnosis of VUR. In addition, ceVUS is a radiation-free examination method with a low risk profile that offers an exceptional diagnostic tool in the diagnostic clarification of recurrent urinary tract infections with the suspected diagnosis of VUR and should also be included in the consideration of a diagnosis next to the established VCUG, especially in younger children.

## 1. Introduction

Vesicoureteral reflux (VUR) describes a common urological pediatric anomaly and occurs according to the literature in 1–2% of the pediatric population [1,2,3,4,5]. VUR is defined as the retrograde flow of urine from the bladder to the upper urinary tract [1,2,6]. The prevalence of VUR in children with documented upper urinary tract infection (UTI) ranges from 30–40% and in approximately one-third of children with a febrile infection in infancy; the underlying etiology was bladder-bowel dysfunction (BBD) [1,7,8,9,10]. Undiagnosed and recurrent VUR may lead to pyelonephritis, renal scarring, arterial hypertension or can even end in kidney failure [1,2,3]. The underlying pathogenesis of VUR in early childhood remains unclear, but a congenital anomaly at the vesicoureteral junction in the context of an abnormal embryological development seems to be the main reason for the emergence of VUR [11]. A strong genetic component could recently be postulated, while having a prevalence of VUR in siblings in 27.4% and in children with affected parents in 35.7% [12]. A specific gene responsible for VUR has, however, not yet been found [3,13]. The ratio of women to men differs along the literature. While one study reported a prevalence in children under 6 months with a 3:1 ratio and a balanced ratio from the age of 21 months [14], another study showed a 9:1 ratio between women and men [15].

In the clinical setup, imaging modalities, such as voiding cystouretherography (VCUG) or contrast-enhanced voiding urosonography (ceVUS), play an important role in the diagnosis of VUR. In addition to its radiation-free nature, the ceVUS also offers high sensitivity and specificity in comparison to the VCUG, as well as a good diagnostic evaluation of the urethra, which makes ceVUS interesting in the diagnostic cascade for the exclusion or quantification of VUR [6,16,17]. Compared to VCUG, ceVUS shows excellent diagnostic sensitivity of up to 100% in detecting VUR [18,19,20]. In order to visualize and diagnose VUR, contrast medium is injected into the bladder via a urinary catheter. *SonoVue^®^*, approved by the U.S. Food and Drug administration (FDA) and the European Medicine agency (EMA) for the study of pediatric urinary tract to detect VUR, is the most commonly used contrast agent and has an outstanding safety profile in adults and children, as well as in initial studies in pregnant women [16,21,22,23,24,25,26].

While ceVUS itself can be used in the intraoperative setting for real-time evaluation of therapeutic efficacy in endoscopic procedures, the European Association of Urology continues to regard the VCUG as the gold standard in the diagnosis and graduation of VUR [27,28]. Including the EFSUMB guidelines, ceVUS is particularly recommended with regard to the outcome after conservative or surgical treatment, in the screening of high-risk patients such as siblings or in patients after a kidney transplantation [29]. Compared to the VCUG, the ceVUS offers the advantages of an exceptionally low risk profile with regard to the administration of contrast medium (SonoVue^®^); it is performed without radiation exposure to the patient and the accompanying person (in the case of small children) and the examination can be performed in a relaxed atmosphere and in a supine position directly at the patient’s bedside. In addition to these benefits, studies already show that ceVUS can be considered at least as equivalent to VCUG for the diagnosis of VUR [17,19,30,31,32].

Referring to the RIVUR study, continuous antibiotic administration in conservative therapeutic management shows a halving of the risk of febrile recurrence compared to the placebo. In particular, children suffering from BBD at baseline or children whose index infection was febrile benefited from prophylactic antimicrobial therapy with a reduction in recurrence in 80% and 60% of cases, respectively [15]. In addition to the positive results of antibiotic therapy, there are also critical voices that postulate an increase risk in bacterial resistance. In addition, early antibiotic administration in animal studies showed an increased risk of adiposity, while prenatal or early antibiotics in other studies showed a slightly increased risk of childhood asthma [8,33,34]. Surgical therapy in the setting of VUR also remains controversial in some aspects, with the goal of focusing on those patients who will not have a spontaneous remission of symptoms and those who are at an increased risk of pyelonephritis or renal scarring [35]. Endoscopic therapy was first described in 1981 by Matouschek et al. and was later popularized by O’Donnell and Puri in 1984 when they invented the subureteric Teflon injection method (STING) [36,37]. The STING procedure involves an injection approximately 2–3 mm distal to the ureterovesical junction at the 6-o’clock-position with placement of the needle in the submucosal plane [38,39]. The hydrodistention-implantation technique (HIT) method, a modified STING method, was introduced by Kirsch et al. in 2004 and describes an injection in the submucosa of the mid/distal ureteral tunnel [39,40,41]. Based on a large meta-analysis comparing the two treatment options after one endoscopic injection, the HIT method showed a better overall success rate with a percentage of 82.5% compared to 71.4% when using the STING method [39]. Another option that has become increasingly popular in recent years is the Double-HIT method, in which a more proximally and more distally ureteral DEFLUX injection is performed [8,38,42]. Endoscopic treatment methods are being countered by laparoscopic and open surgical techniques, which are superior to endoscopic therapy with a success rate of up to 95–98% [33,38,43]. The laparoscopic ureteral reimplantation was first described by Atala et al. in 1993. The Lich-Gregoir method describes an extravesical ureteral reimplantation, while in 2001, Gill et al. introduced the first intravesical ureteral reimplantation [44,45].

## 2. Materials and Methods

In the period from July 2016 to May 2020, 49 patients where referred to the Radiology Department and could retrospectively be enrolled in the present study. This group of patients included 45 female and 2 male patients. The age distribution ranged from 5 months to 60 years with a distribution from under to over 18 years of 4:1. In order to achieve a sufficient image quality, the examinations were made in the supine position. 

Before being included in the study, a detailed medical consultation about all potential risks took place and oral and written informed consent was obtained from every patient. After ceVUS was performed, the patient data and the imaging files were stored in the inhouse achieving system to allow further analysis and a detailed interpretation of the gained data.

All examinations were performed by a single experienced radiologist (EFSUMB level 3) with a professional experience since 2000. The examinations were carried out using the latest ultrasound equipment available up-to-date CEUS protocols at the time of the examination (Philips Ultrasound iU22, EPIQ 7, Seattle, Washington, DC, USA; Samsung RS 80, Seoul, Korea). The contrast medium used was SonoVue (Bracco, Milan, Italy), a second-generation blood-pool contrast agent with the characteristics of a purely intravascular distribution pattern. SonoVue has been approved by the U.S. Food and Drug administration (FDA) and the European Medicine agency for the study of the pediatric urinary tract to detect VUR [15,22]. To avoid an early destruction of the applied microbubbles, a low mechanical index of <0.2 has been used.

In advance, the urological colleagues place a urinary catheter to safely administer contrast medium. To ensure good contrast of the urinary tract, the bladder was filled with a solution of 50 mL 0.9% saline with an included amount of 0.3 mL SonoVue. Depending on the age of the patient and the size of the bladder, 1–3 injections of the diluted contrast medium had to be applied to maintain a good balance between a too low and too high amount of contrast medium. An insufficient amount of injected microbubbles can lead to a reduced delimitation of the cavity border, while too high volumes of contrast medium may lead to a pooling of the microbubbles due to the limited ability of free movement or distribution [46]. 

**Figure 1:**
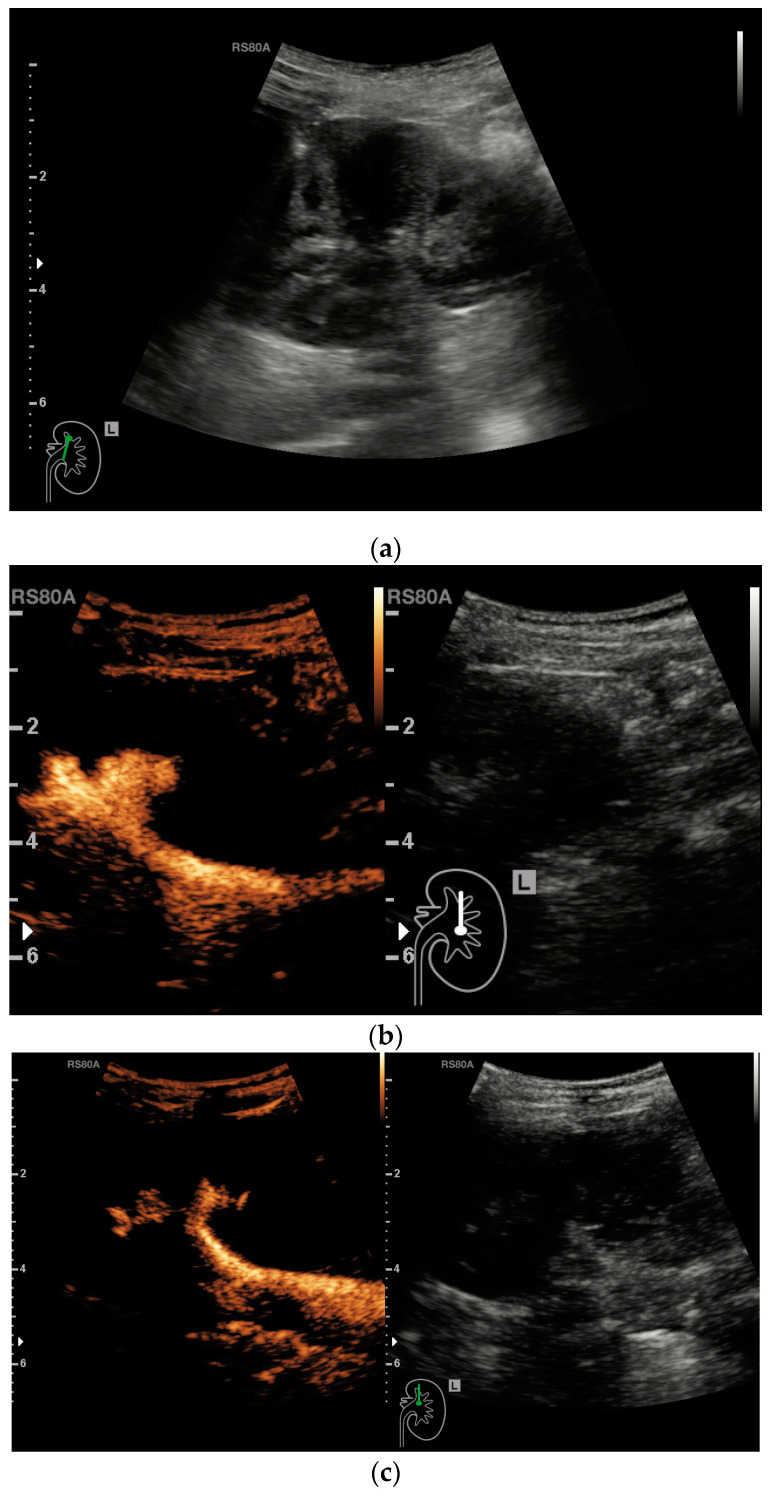
Month year old girl with recurrent upper urinary tract infections showing a moderate dilatation of the renal pelvis and a slight dilatation of the calyces in conventional B-mode (**a**). After the administration of contrast medium, a vesicoureteral reflux with a clear retrograde enhancement of the dilatated renal pelvis and a slight enhancement of the dilatated calyces can be seen (**b**,**c**). These sonomorphological findings indicate vesicoureteral reflux grade II.

In the clinical examination, VUR was categorized into 5 subcategories. If CEUS only showed a reflux in the non-dilated ureter, it implied a reflux Grade I, while in Grade II, the renal pelvis and calyces were also involved (Figure 1). 

**Figure 2:**
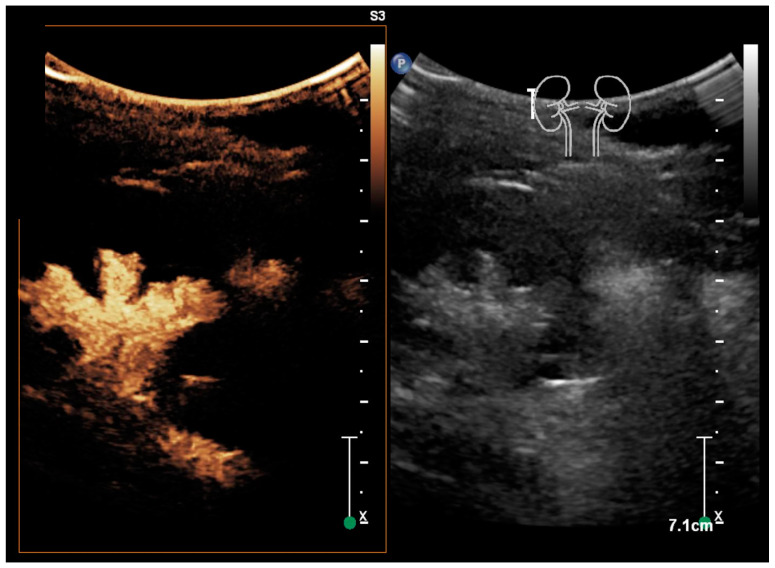
One year-old girl with recurrent upper urinary tract infections in her medical history showing a marked dilatation of the renal pelvis and calyces in conventional B-mode (right side) with a concomidant marked retrograde enhancement of the pyelo-ureteral unit after the administration of SonoVue© (left side). These sonomorphological findings indicate vesicoureteral reflux grade III.

Grade III furthermore describes a mild to moderate dilatation of the ureter and renal pelvis, whereas no or just a minimal blunting of the fornices can be seen (Figure 2). A moderate dilatation and tortuosity of the ureter, renal pelvis and calyces indicate the pathological changes in Grad IV, while Grad V includes a gross dilatation and tortuosity of the ureter, the renal pelvis and the calyces with an accompanying loss of the papillary impressions [43,47,48].

## 3. Results

In the time period from July 2016 to May 2020, 49 patients underwent a ceVUS to assess VUR. 95.9% of the patients were female (*n* = 47), whereas only two male patients were included in the patient cohort. Additionally, four patients had a duplex kidney with a bifid ureter, whereby in two patients the right and in two patients the left kidney was affected. For the four patients, three of them showed a striking sonomorphological finding. In summary, 102 pyelo-ureteral units (PPU) were investigated. The median patient age was 7.9 years with a mean patient age of 11.4 years. The patient cohort has an age distribution between 5 months and 60 years, but 38 out of the 49 patients were under 18 years (77.6%).

Among the patients examined, slightly more than half of the patients had four or more UTIs (*n* = 32, 65.3%). The second largest patient group had a single UTI in their records (20.4%; *n* = 10) (Table 1).

After the administration of contrast medium via the urinary catheter, a graduation of the reflux was performed according to the common classification, which specifies five different subtypes. Under closer examination of the patients with a left-sided conspicuous finding (*n* = 14), five patients could be classified with VUR grade II and three patients could be classified with VUR grade III. Another two patients showed sonomorphological signs of VUR grade I-II, and four patients with VUR grade II–III. The eight patients with a pathological right-sided finding could each be classified in the dynamic examination with VUR grade II (*n* = 3), VUR grade III (*n* = 3) and II–III (*n* = 2), while the two patients with bilateral reflux showed reflux grade I-II and II on the left side and reflux grade III on the right side (Table 2). VUR grade I or grade IV and V could not be found in any of the included patients.

If the number of febrile UTIs according to Table 1 is compared to the severity of VUR, the patient cohort with a UTI of ≥4 (*n* = 32) reveals sonomorphological findings that are conspicuous for VUR in almost 50% of the cases (*n* = 15; 46.9%). Among the fifteen affected patients, a shift to a higher graduation was observed. Five patients had VUR grade II (33.3%), four patients had VUR grade II–III (26.7%), while six patients had a reflux grade III (40%).

If the patients with an age of older than 18 years are excluded from this subgroup, the group size within the patients with more ≥4 UTIs is reduced by 15 with 17 remaining patients in this cohort. Among these 17 patients, nine patients have an inconspicuous finding in ceVUS (52.9%), while three patients had VUR grade II (17.6%), two patients had VUR grade II–III (11.8%) and three patients had grade III (17.6%).

To deal with the value of ceVUS in terms of clinical relevance, a closer look at the further clinical course and outcome of the patients is needed. Of the 22 sonomorphologically affected patients with VUR, 17 patients underwent surgery (90.9%), three patients received antibiotic therapy (7.1%) while two patients had no further documentation about their further therapeutic course. Of the 17 surgically treated patients, of whom 2 patients were bilaterally affected (19 PUU), the HIT/STING method was applied six times and the laparoscopic Lich-Gregoir method twelve times (Table 3). One patient with a duplex kidney and ectopic ureter opening into the urethra was treated by ureteroureterostomy therapy.

## 4. Discussion

As a common pediatric urological anomaly, VUR requires timely diagnosis and treatment to prevent irreparable kidney damage. In order to assess the diagnostic relevance of ceVUS, it is necessary to take a close look at the knowledge gained from the ultrasound examination and the outcome. Of the 49 patients included, none of the patients were indicated for additional VCUG. In all 27 sonomorphologically inconspicuous patients, no surgical treatment was performed after a monitoring period of 2 months to 4 years (mean value 1.8 years), which demonstrates the high specificity of ceVUS. In combination with the subsequent surgical procedure in the present patient population, intraoperative confirmation of VUR was seen in all 17 cases that were positively diagnosed by ceVUS. Only in one patient where the left kidney was initially diagnosed as inconspicuous had to undergo a surgical treatment. When looking at the included patients and correlating it to intraoperative findings, a sensitivity of 95.7% and a specificity of 100% is shown which is consistent with other study results [49,50]. Considering the subdivision of the patients into four or more UTIs, there is no significant difference in severity in analogy to the overall cohort. A direct correlation between the number of febrile UTIs and the severity of VUR cannot be drawn (compare Table 2 and Table 4). Rather, it seems likely that patients with an increased number of febrile UTIs also had an association with other disorders, such as BBD. Regarding the cohort, a follow-up procedure after outpatient treatment were necessary after the initial endoscopic intervention in two patients. In summary, the endoscopic treatment in our patient cohort is equivalent to a success rate of 90.9%, which agrees with the common literature [36,51,52]. This aspect indicates a good selection of patients for a specific surgical procedure, a superiority of ceVUS compared to VCUG in terms of outcome cannot, of course, be concluded.

In a study by Piskunowicz et al. in 2016, the NPV was 97% which is similar to the diagnostic value of VCUG [53]. Notwithstanding, on the basis of the 2012 and 2015 published Guidelines of the European Association of Urology (EAU) and in the American Urological Association Guidelines, the VCUG still remains the gold standard [1,26,54]. In addition to the convincing results of ceVUS in VUR, it should not be neglected that the safety profile of the study is remarkably good. In comprehensive studies, it could be demonstrated that the contrast medium used (SonoVue^®^), which has been approved by the FDA and EMA, only had minor side effects in 3.7% of cases. These complications, however, were usually associated to the bladder catheterization which, however, must be performed in both VCUG and ceVUS patients [18,21,23,25]. In addition, it should be considered that ceVUS does not expose the patient and possible accompanying persons to radiation, the risk profile of the contrast agent is negligible and thus an examination can be performed at any time without major medical preparation. The possibility of performing the examination directly at the patient’s bedside can also generate a relaxed atmosphere for the patient, which has a positive effect on the doctor-patient relationship, especially for small children. In terms of cost-effectiveness, ceVUS is also superior to VCUG [20,55]. 

## 5. Conclusions

In the underlying patient cohort of 49 patients, ceVUS offers a sensitivity of 95.7%, a specificity of 100%, a PPV of 100% and an NPV of 98.6% in detecting VUR with regard to a further surgical intervention. Taking into account the many advantages, e.g., the exceptional compatibility of the contrast medium or the lack of radiation exposure of the patient and accompanying person compared to VCUG, ceVUS should find more widespread use in the daily routine in the exclusion or conformation of VUR.

## Figures and Tables

**Table 1:** Number of upper urinary tract infections in relation to the number of patients affected.

Number of Upper Urinary Tract Infections	Number of Patients	Percentage Distribution
0	4	8.2%
1	10	20.4%
2	2	4.1%
3	1	2.0%
4	5	10.2%
>4	27	55.1%
Total number of patients	49	100%

**Table 2:** Differentiation into the individual grades and subdivision of the affected side of the sonomorphologically affected patients.

	Number of Patients (*n* = 22) Depending on the Sonomorphologically Conspicuous Side			
	Left Side	Right Side	Both Sides	
			Left	Right
Grade I	-	-	-	-
Grade I–II	2	-	1	-
Grade II	5	3	1	-
Grade II–III	4	2	-	-
Grade III	3	3	-	2
Total number of patients	14	6	2	

**Table 3:** Therapeutic course after performing ceVUS.

	Endoscopic Treatment	Laparoscopic Surgery Procedure	Conservative Treatment	No Further Records
Patients	17		3	2
affected kidneys	6	12	1	1
patients < 18 years	3	6	2	1
patients > 18 years	3	6	1	1
Admission in 2018	4	1	1	1
Admission in 2019	1	4	1	1
Admission in 2020	1	7	1	-

**Table 4:** Differentiation of the different VUR grades in patients >4 UTI both in the total cohort and in patients younger than 18 years of age.

	Number of Patients with ≥4 UTIs Depending on the Sonomorphologically Conspicuous Side		Number of Patients with ≥4 UTIs Depending on the Sonomorphologically Conspicuous side/Younger than 18 Years	
	Left Side	Right Side	Left Side	Right Sight
no evidence of VUR	17		9	
Grade II	4	1	2	1
Grade II–III	3	1	2	-
Grade III	2	4	1	2
Total number of patients	32		17	

## Data Availability

The data presented in this study are available on request from the corresponding author. The data are not publicly available due to privacy.
